# Supplementary material for: FBXO7 ubiquitinates PRMT1 to suppress serine synthesis and tumor growth in hepatocellular carcinoma
Source: Nat Commun. 2024 Jun 5;15:4790. doi: 10.1038/s41467-024-49087-2 (PMC11153525; doi:10.1038/s41467-024-49087-2)
Supplement: Supplementary file 1 — Supplementary Information [file 41467_2024_49087_MOESM1_ESM.pdf]

## Supplementary Information

# FBXO7 Ubiquitinates PRMT1 to Suppress Serine Synthesis and Tumor Growth in Hepatocellular Carcinoma

Li Luo, Xingyun Wu, Jiawu Fan, Lixia Dong, Mao Wang, Yan Zeng, Sijia Li, Wenyong Yang, Jingwen Jiang, Kui Wang

## Contents

**Supplementary Figures 1-7**

**Supplementary Table 1**

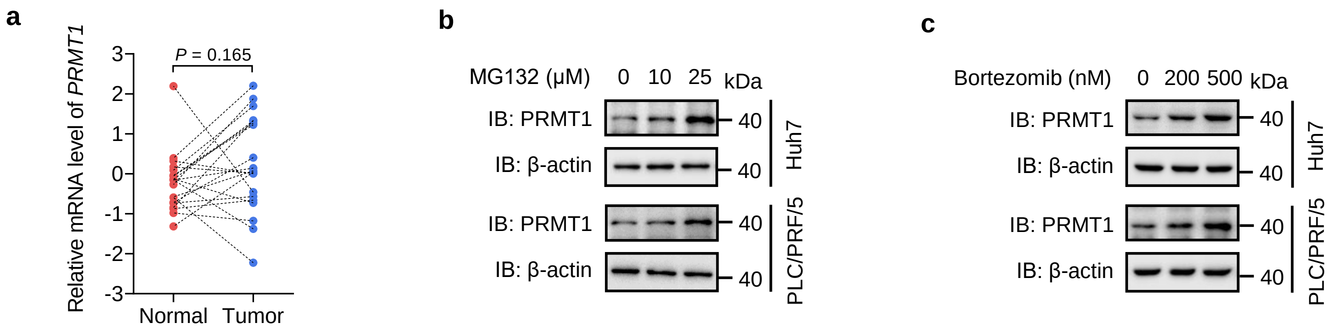


**Supplementary Figure 1. Decreased proteasomal degradation, but not elevated mRNA level, may contributes to PRMT1 upregulation in HCC.**

**a** The relative mRNA level of *PRMT1* in HCC tissues or paired adjacent normal liver tissues determined by qPCR analysis ($n$ = 20 samples). Data are presented as mean ± SD. Statistical analysis was performed using the paired two-tailed Student's t-test. **b** Immunoblotting analysis of PRMT1 (anti-PRMT1 antibody: Abcam, ab190892) in Huh7 and PLC/PRF/5 cells treated with MG132 at indicated concentrations for 6 h. The immunoblotting experiments were repeated three times with similar results. **c** Immunoblotting analysis of PRMT1 (anti-PRMT1 antibody: Abcam, ab190892) in Huh7 and PLC/PRF/5 cells treated with bortezomib at indicated concentrations for 5 h. The immunoblotting experiments were repeated three times with similar results. Source data are provided as a Source Data file.

**a**

| Proteins | Ratio (FLAG-PRMT1/Vector) | No. Unique peptides |
|---|---|---|
| PRPH | 522.78 | 10 |
| TUSC1 | 226.47 | 6 |
| **FBXO7** | **184.09** | **7** |
| NEFL | 176.61 | 11 |
| TAF15 | 140.66 | 15 |
| ANGEL2 | 138.18 | 12 |
| RAMAC | 133.72 | 6 |
| HNRNPH3 | 121.27 | 10 |
| RBFOX2 | 110.34 | 6 |
| LSM14A | 109.89 | 13 |

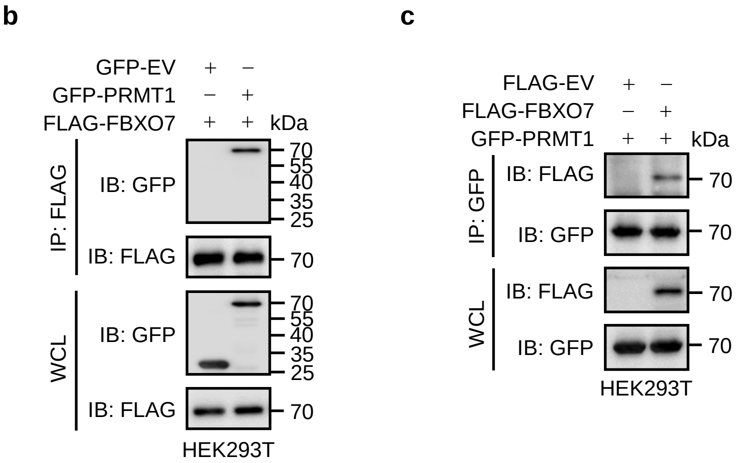


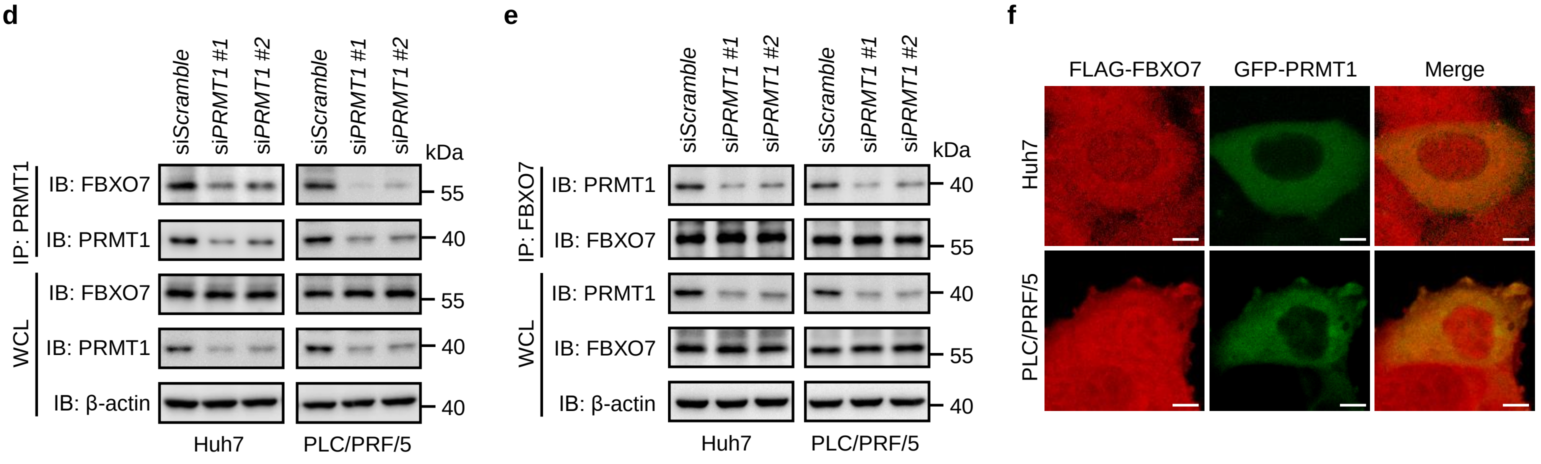


**Supplementary Figure 2. FBXO7 is an interacting partner of PRMT1.**

**a** FLAG-PRMT1 was immunopurified by FLAG beads in Huh7 cells stably expressing FLAG-PRMT1. Immunoprecipitates were then digested by trypsin and subjected to LC-MS/MS for label-free quantitative proteomics analysis. **b, c** Reciprocal co-IP analysis of GFP-PRMT1 and FLAG-FBXO7 in HEK293T cells. The immunoblotting experiments were repeated three times with similar results. **d, e** Reciprocal co-IP analysis of PRMT1 and FBXO7 in Huh7 and PLC/PRF/5 cells transfected with siRNA targeting *PRMT1*. The immunoblotting experiments were repeated three times with similar results. **f** Immunofluorescent analysis of the colocalization of FLAG-FBXO7 with GFP-PRMT1 in Huh7 and PLC/PRF/5 cells. Scale bars: 10 μm. Source data are provided as a Source Data file.

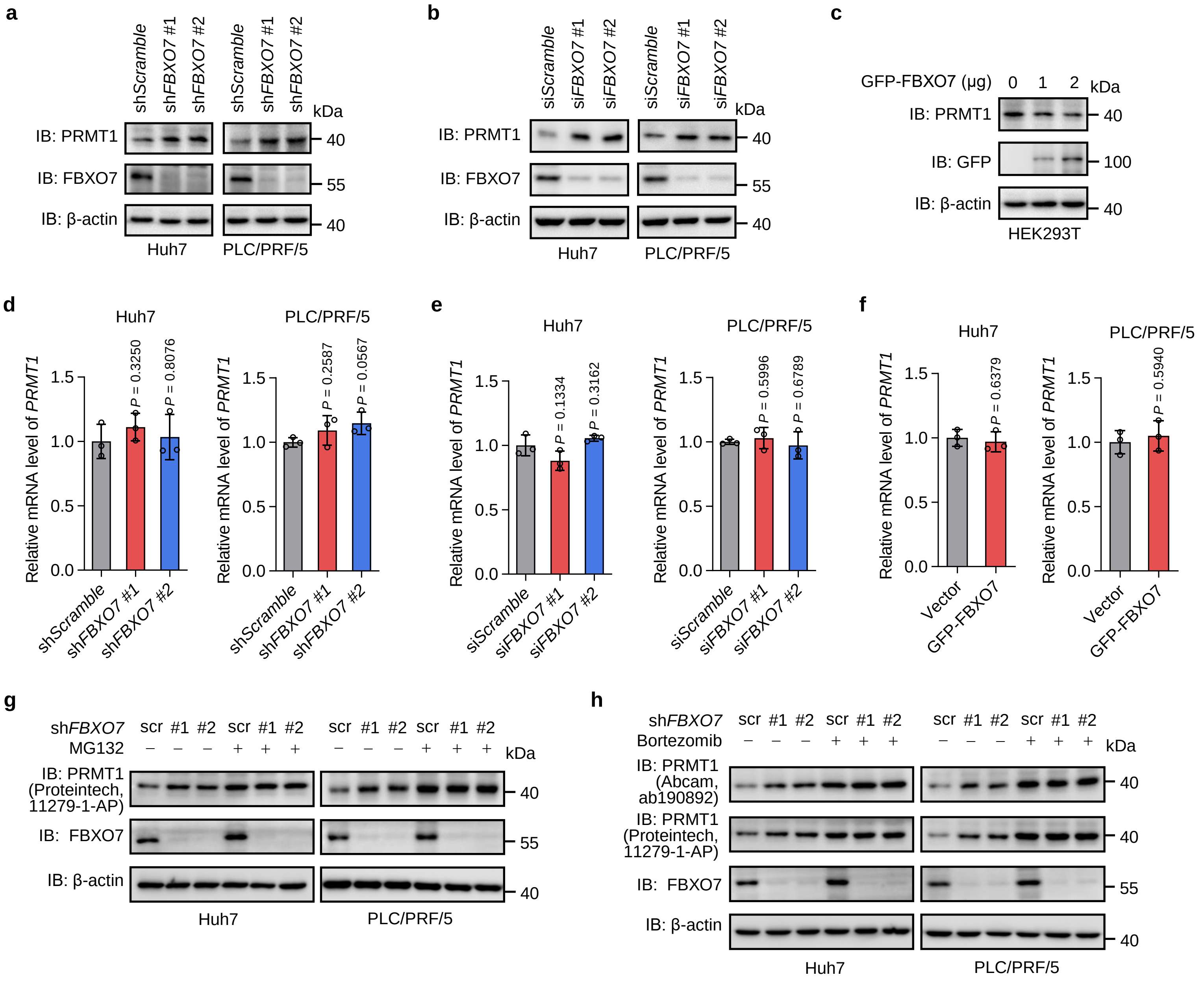


**Supplementary Figure 3. FBXO7 downregulates the protein level of PRMT1 by promoting its proteasomal degradation, but has no obvious effect on the mRNA level of *PRMT1*.**

**a, b** Immunoblotting analysis of PRMT1 and FBXO7 in *FBXO7* KD cell using shRNA (**a**) or siRNA (**b**) targeting *FBXO7*. The immunoblotting experiments were repeated three times with similar results. **c** Immunoblotting analysis of PRMT1 and GFP-FBXO7 in cells overexpressing GFP-FBXO7. The immunoblotting experiments were repeated three times with similar results. **d, e** The relative mRNA level of *PRMT1* in *FBXO7* KD cells using shRNA (**d**) or siRNA (**e**) targeting *FBXO7*. Data are presented as the mean ± SD ($n = 3$ independent experiments). Statistical analysis was performed using the two-tailed Student's *t*-test. **f** The relative mRNA level of *PRMT1* in GFP-FBXO7-oversexpressing Huh7 and PLC/PRF/5 cells. Data are presented as the mean ± SD ($n = 3$ independent experiments). Statistical analysis was performed using the two-tailed Student's *t*-test. **g** Immunoblotting analysis of PRMT1 (anti-PRMT1 antibody: Proteintech, 11279-1-AP) and FBXO7 in *FBXO7* KD cells treated with or without MG132 (25 μM, 6 h). The immunoblotting experiments were repeated three times with similar results. **h** Immunoblotting analysis of PRMT1 (anti-PRMT1 antibody: Abcam, ab190892; Proteintech, 11279-1-AP) and FBXO7 in *FBXO7* KD cells treated with bortezomib (500 nM, 5 h). The immunoblotting experiments were repeated three times with similar results. Source data are provided as a Source Data file.

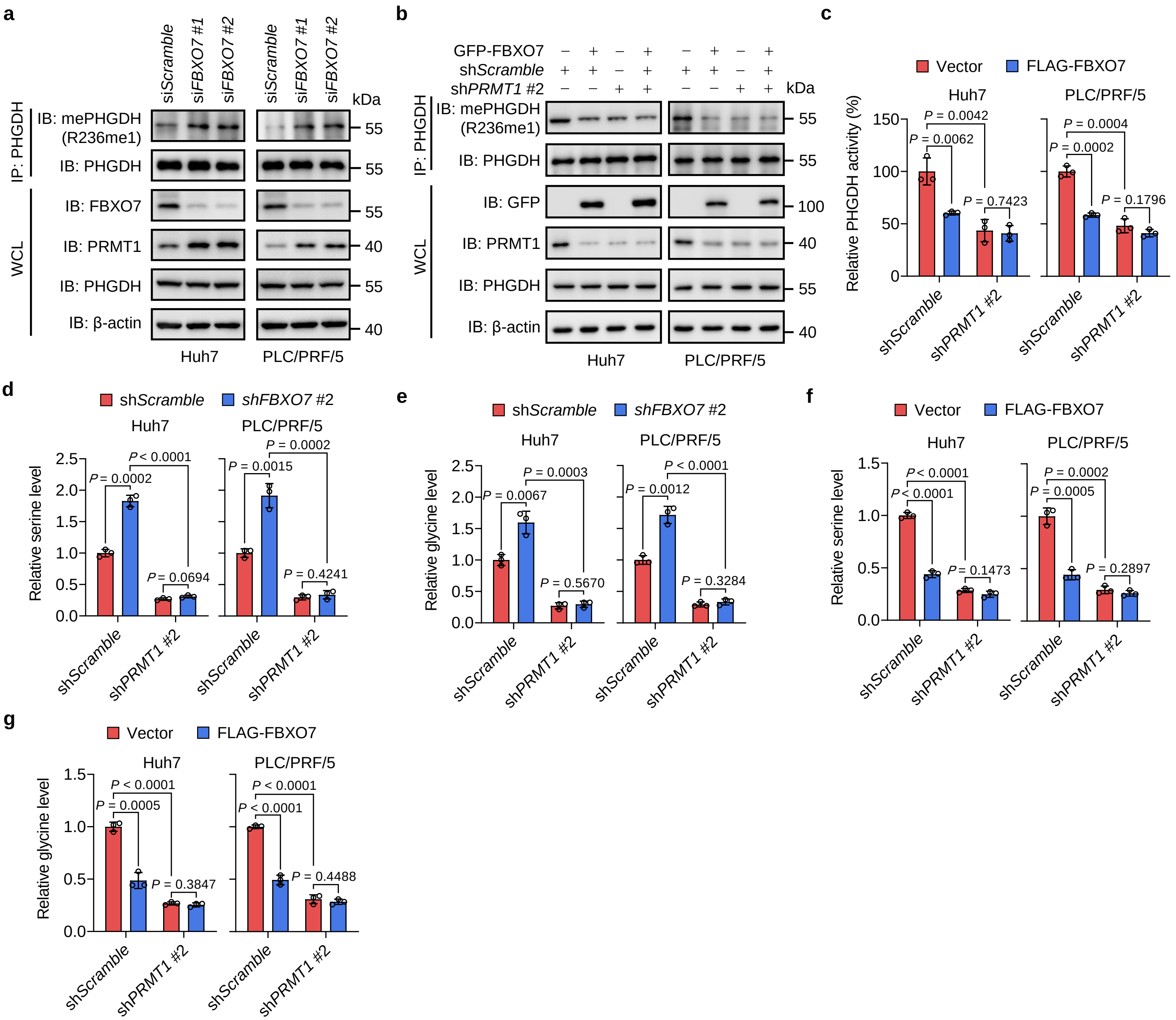


**Supplementary Figure 4. FBXO7 inhibits PHGDH methylation, PHGDH activity, and serine synthesis by downregulating PRMT1 in HCC cells.**

**a** PHGDH was immunoprecipitated in HCC cells transfected with *FBXO7* siRNA, followed by immunoblotting with indicated antibodies. The immunoblotting experiments were repeated three times with similar results. **b** PHGDH was immunoprecipitated in GFP-FBXO7-overexpressing and/or *PRMT1* KD cells, followed by immunoblotting with indicated antibodies. The immunoblotting experiments were repeated three times with similar results. **c** Endogenous PHGDH was immunoprecipitated in FLAG-FBXO7-overexpressing and/or *PRMT1* KD cells, followed by measurement of PHGDH activity. Data are presented as the mean ± SD ($n = 3$ independent experiments). Statistical analysis was performed using the two-tailed Student's *t*-test. **d, e** Total serine (**d**) and glycine (**e**) levels in *FBXO7* and/or *PRMT1* KD cells cultured in serine- and glycine-depleted (-SG) medium. Data are presented as the mean ± SD ($n = 3$ independent experiments). Statistical analysis was performed using the two-tailed Student's *t*-test. **f, g** Total serine (**f**) and glycine (**g**) levels in *PRMT1* KD and/or FLAG-FBXO7-overexpressing cells cultured in -SG medium. Data are presented as the mean ± SD ($n = 3$ independent experiments). Statistical analysis was performed using the two-tailed Student's *t*-test. Source data are provided as a Source Data file.

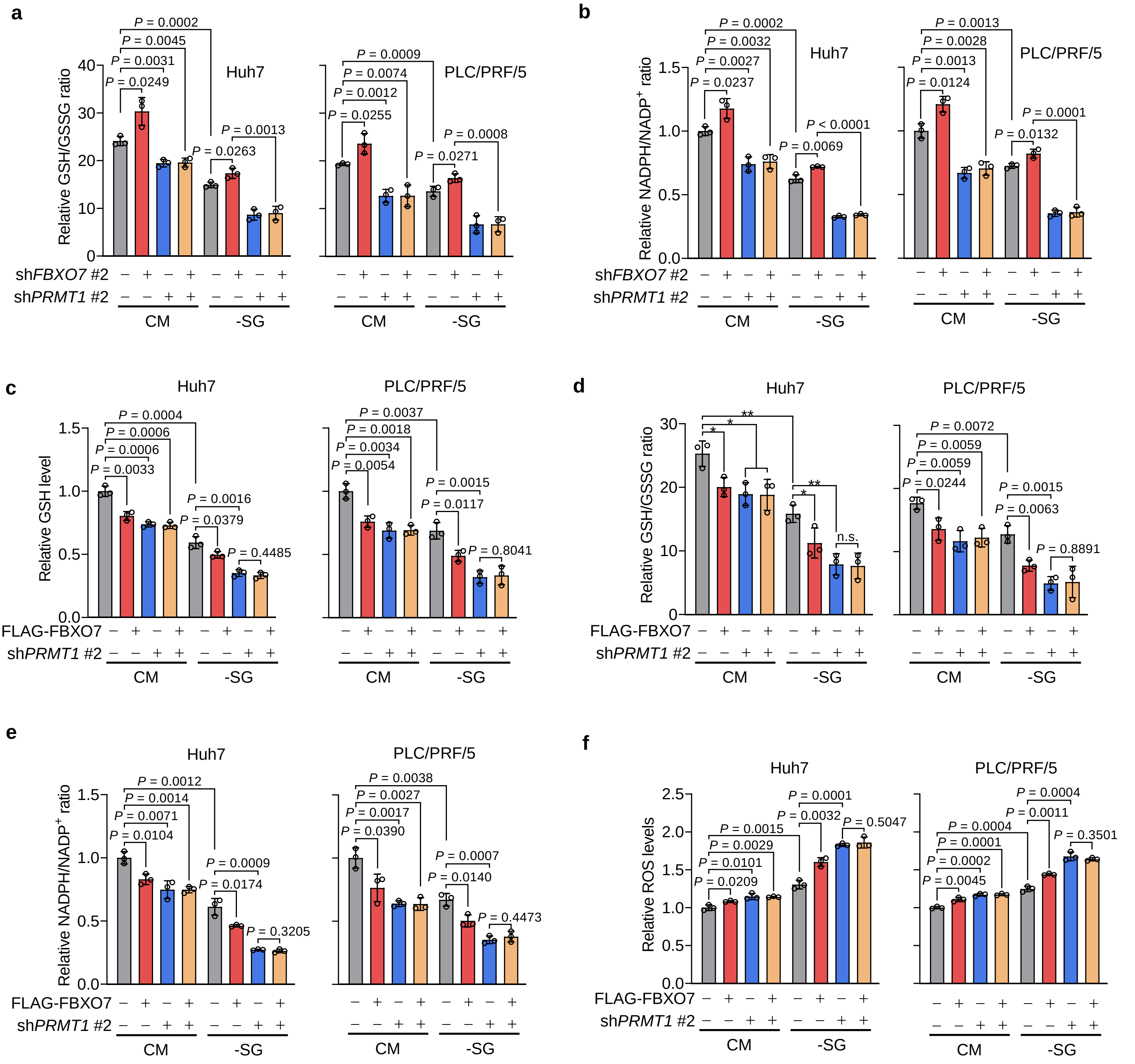


**Supplementary Figure 5. FBXO7 promotes oxidative stress by downregulating PRMT1 in HCC cells.**

**a, b** GSH/GSSG ratio (**a**) and NADPH/NADP+ ratio (**b**) in *FBXO7* and/or *PRMT1* KD cells cultured in complete (CM) or serine/glycine-depleted (-SG) medium. Data are presented as the mean ± SD ($n = 3$ independent experiments). Statistical analysis was performed using the two-tailed Student's *t*-test. **c-e** GSH level (**c**), GSH/GSSG ratio (**d**), and NADPH/NADP$^+$ ratio (**e**) in *PRMT1* KD and/or FLAG-FBXO7-overexpressing cells cultured in CM or -SG medium. Data are presented as the mean ± SD ($n = 3$ independent experiments). Statistical analysis was performed using the two-tailed Student's *t*-test. **f** ROS levels in *PRMT1* KD and/or FLAG-FBXO7-overexpressing cells cultured in CM or -SG medium. Data are presented as the mean ± SD ($n = 3$ independent experiments). Statistical analysis was performed using the two-tailed Student's *t*-test. Source data are provided as a Source Data file.

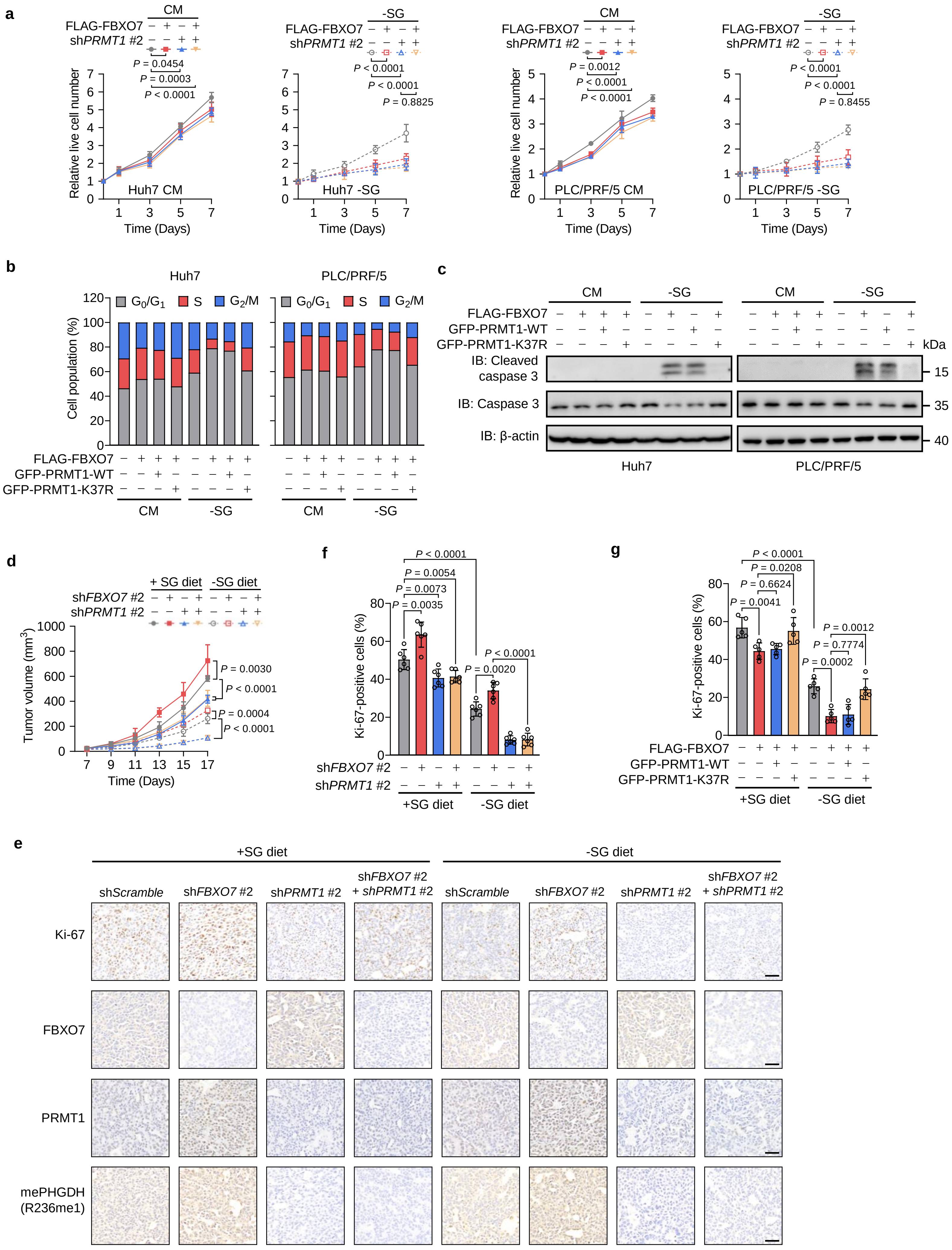
a
CM
FLAG-FBXO7
shPRMT1 #2
P = 0.0454
P = 0.0003
P < 0.0001
Relative live cell number
Huh7 CM
Time (Days)
-SG
P < 0.0001
P < 0.0001
P = 0.8825
Huh7 -SG
P = 0.0012
P < 0.0001
P < 0.0001
PLC/PRF/5 CM
P < 0.0001
P < 0.0001
P = 0.8455
PLC/PRF/5 -SG
b
Huh7
PLC/PRF/5
G0/G1
S
G2/M
Cell population (%)
FLAG-FBXO7
GFP-PRMT1-WT
GFP-PRMT1-K37R
CM
-SG
c
CM
-SG
FLAG-FBXO7
GFP-PRMT1-WT
GFP-PRMT1-K37R
kDa
IB: Cleaved caspase 3
IB: Caspase 3
IB: β-actin
15
35
40
Huh7
PLC/PRF/5
d
+ SG diet
-SG diet
shFBXO7 #2
shPRMT1 #2
Tumor volume (mm3)
P = 0.0030
P < 0.0001
P = 0.0004
P < 0.0001
Time (Days)
f
P < 0.0001
P = 0.0054
P = 0.0073
P = 0.0035
P < 0.0001
P = 0.0020
Ki-67-positive cells (%)
shFBXO7 #2
shPRMT1 #2
+SG diet
-SG diet
g
P < 0.0001
P = 0.0208
P = 0.6624
P = 0.0041
P = 0.0012
P = 0.7774
P = 0.0002
Ki-67-positive cells (%)
FLAG-FBXO7
GFP-PRMT1-WT
GFP-PRMT1-K37R
+SG diet
-SG diet
e
+SG diet
-SG diet
shScramble
shFBXO7 #2
shPRMT1 #2
shFBXO7 #2 + shPRMT1 #2
Ki-67
FBXO7
PRMT1
mePHGDH (R236me1)

**Supplementary Figure 6. FBXO7 suppresses the growth and proliferation, and induces apoptosis in HCC cells by downregulating PRMT1.**

**a** Growth rates of *PRMT1* KD and/or FLAG-FBXO7-overexpressing cells grown in complete (CM) or serine/glycine-depleted (-SG) medium. Data are presented as the mean ± SD ($n = 5$ independent experiments). Statistical analysis was performed using the two-way ANOVA with Bonferroni correction. **b** Cell cycle distribution of Huh7 and PLC/PRF/5 cells overexpressing FLAG-FBXO7 and GFP-PRMT1-WT or K37R cultured in CM or -SG medium. **c** Immunoblotting analysis of cleaved caspase 3 and caspase 3 in cells overexpressing FLAG-FBXO7 and GFP-PRMT1-WT or K37R cultured in CM or -SG medium. The immunoblotting experiments were repeated three times with similar results. **d** The volume of tumor xenografts from nude mice inoculated with *FBXO7* and/or *PRMT1* KD Huh7 cells and fed with a +SG or -SG diet. Data are presented as the mean ± SD ($n = 6$ mice). Statistical analysis was performed using the two-way ANOVA with Bonferroni correction. **e** Representative images of IHC staining for Ki-67, FBXO7, PRMT1, and mePHGDH (R236me1) in tumor xenografts in **d**. **f** Quantitative analysis of IHC staining for Ki-67 in **e**. Data are presented as the mean ± SD ($n = 6$ mice). Statistical analysis was performed using the two-tailed Student's *t*-test. **g** Quantitative analysis of IHC staining for Ki-67 of tumor xenografts from nude mice inoculated with Huh7 cells overexpressing FLAG-FBXO7 and GFP-PRMT1-WT or K37R and fed with a +SG or -SG diet. Data are presented as the mean ± SD ($n = 5$ mice). Statistical analysis was performed using the two-tailed Student's *t*-test. Source data are provided as a Source Data file.

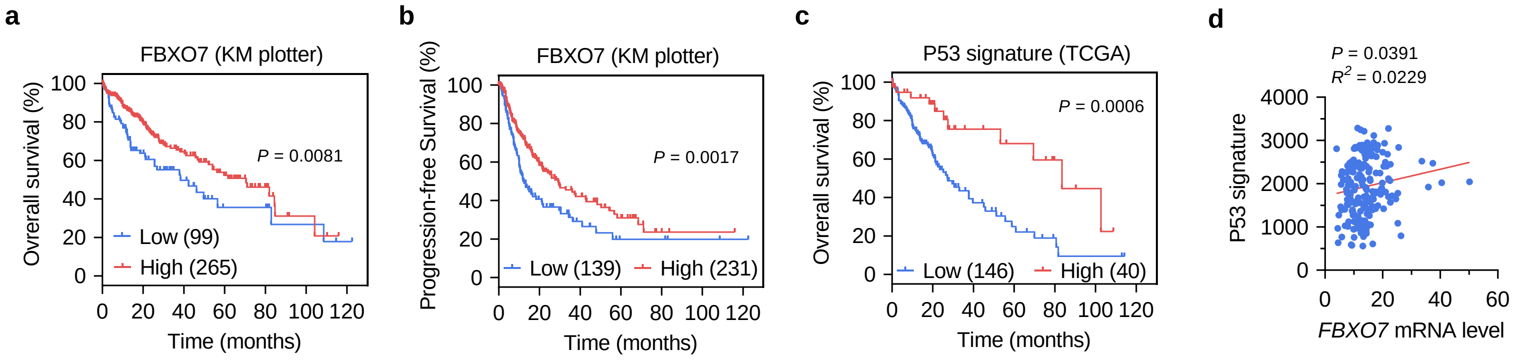


**Supplementary Figure 7. FBXO7 expression positively correlates with p53 signature and HCC patient survival. a, b** Overall survival (**a**) and progression-free survival (**b**) based on FBXO7 level of HCC patients in KM plotter database (*n* = 364 and 370 samples, respectively). Statistical analysis was performed using the two-sided log-rank test. **c** Overall survival of HCC patients based on p53 signature (*n* = 186 samples) in TCGA database. **d** Pearson correlation test analyzing the relationship between *FBXO7* mRNA level and p53 signature in HCC tissues (*n* = 186 samples). Source data are provided as a Source Data file.

**Supplementary Table 1. Clinical characteristics of HCC patients.**

| Characteristics | HCC patients (*n* = 45) |
|---|---|
| Age, year | |
| Median (range) | 53 (35-76) |
| Gender, *n* (%) | |
| Female | 11 (24.4) |
| Male | 34 (75.6) |
| Tumor size, cm (%) | |
| ≤ 5 | 14 (31.1) |
| > 5 | 31 (68.9) |
| TNM stage, *n* (%) | |
| I | 8 (17.8) |
| II | 9 (20.0) |
| III | 28 (62.2) |
