# Supplementary material for: FBXO7 ubiquitinates PRMT1 to suppress serine synthesis and tumor growth in hepatocellular carcinoma
Source: Nat Commun. 2024 Jun 5;15:4790. doi: 10.1038/s41467-024-49087-2 (PMC11153525; doi:10.1038/s41467-024-49087-2)
Supplement: Supplementary file 2 — Reporting Summary [file 41467_2024_49087_MOESM2_ESM.pdf]

nature portfolio

Corresponding author(s): Kui Wang

Last updated by author(s): Mar 19, 2024

Reporting Summary

Nature Portfolio wishes to improve the reproducibility of the work that we publish. This form provides structure for consistency and transparency in reporting. For further information on Nature Portfolio policies, see our Editorial Policies and the Editorial Policy Checklist.

Statistics

For all statistical analyses, confirm that the following items are present in the figure legend, table legend, main text, or Methods section.

n/a	Confirmed	
☐	☒	The exact sample size (*n*) for each experimental group/condition, given as a discrete number and unit of measurement
☐	☒	A statement on whether measurements were taken from distinct samples or whether the same sample was measured repeatedly
☐	☒	The statistical test(s) used AND whether they are one- or two-sided<br>*Only common tests should be described solely by name; describe more complex techniques in the Methods section.*
☒	☐	A description of all covariates tested
☐	☒	A description of any assumptions or corrections, such as tests of normality and adjustment for multiple comparisons
☐	☒	A full description of the statistical parameters including central tendency (e.g. means) or other basic estimates (e.g. regression coefficient) AND variation (e.g. standard deviation) or associated estimates of uncertainty (e.g. confidence intervals)
☐	☒	For null hypothesis testing, the test statistic (e.g. *F*, *t*, *r*) with confidence intervals, effect sizes, degrees of freedom and *P* value noted<br>*Give P values as exact values whenever suitable.*
☒	☐	For Bayesian analysis, information on the choice of priors and Markov chain Monte Carlo settings
☒	☐	For hierarchical and complex designs, identification of the appropriate level for tests and full reporting of outcomes
☐	☒	Estimates of effect sizes (e.g. Cohen's *d*, Pearson's *r*), indicating how they were calculated

Our web collection on statistics for biologists contains articles on many of the points above.

Software and code

Policy information about availability of computer code

Data collection	A Q Exactive Plus Orbitrap LC-MS/MS System (ThermoFisher Scientific) was used collect proteomic data.
Data analysis	GraphPad Prism 8.0 software was used for statistical analyses. Proteome Discoverer software (Version 1.3 and 2.4, ThermoFisher Scientific) was used for proteomic analysis.

For manuscripts utilizing custom algorithms or software that are central to the research but not yet described in published literature, software must be made available to editors and reviewers. We strongly encourage code deposition in a community repository (e.g. GitHub). See the Nature Portfolio guidelines for submitting code & software for further information.

Data

Policy information about availability of data

All manuscripts must include a data availability statement. This statement should provide the following information, where applicable:

- Accession codes, unique identifiers, or web links for publicly available datasets
- A description of any restrictions on data availability
- For clinical datasets or third party data, please ensure that the statement adheres to our policy

The proteomics data generated in this study have been deposited in the ProteomeXchange Consortium (http://proteomecentral.proteomexchange.org) via the iProX partner repository with the dataset identifier PXD045015 (https://proteomecentral.proteomexchange.org/cgi/GetDataset?ID=PXD045015) and PXD049069 (https://proteomecentral.proteomexchange.org/cgi/GetDataset?ID=PXD 049069). Source data are provided with this paper.

Research involving human participants, their data, or biological material

Policy information about studies with human participants or human data. See also policy information about sex, gender (identity/presentation), and sexual orientation and race, ethnicity and racism.

Reporting on sex and gender	An HCC cohort containing 45 patients (11 Female, 34 Male) were used in this study. Sex and/or gender of participants was determined based on self-report.
Reporting on race, ethnicity, or other socially relevant groupings	The hepatocellular carcinoma (HCC) tissues used in this study were obtained from West China Hospital, Chengdu, with no consideration of their race, ethnicity, or other socially relevant groupings in the study design.
Population characteristics	The hepatocellular carcinoma (HCC) tissues used in this study were obtained from West China Hospital, Chengdu, with written consent from patients. The detailed patient demographics and clinical characteristics were shown in Supplementary Table 1.
Recruitment	There were no bias on the selection of patients. The samples of patients diagnosed with HCC by immunohistochemical analysis were selected. The normal adjacent tissue samples were obtained from the same patients.
Ethics oversight	Ethics approval was obtained from the Institutional Ethics Committee of Sichuan University.

Note that full information on the approval of the study protocol must also be provided in the manuscript.

Field-specific reporting

Please select the one below that is the best fit for your research. If you are not sure, read the appropriate sections before making your selection.

[x] Life sciences [ ] Behavioural & social sciences [ ] Ecological, evolutionary & environmental sciences

For a reference copy of the document with all sections, see nature.com/documents/nr-reporting-summary-flat.pdf

Life sciences study design

All studies must disclose on these points even when the disclosure is negative.

Sample size	The sample sizes were determined based on standard protocols in the field (Yajuan Zhang et al., J Clin Invest. 2021, PMID: 34720086; Chao Wang et al., Cell Rep. 2020, PMID: 32783943; Wang et al., Nat Commun. 2023, PMID: 36823188; Mylène Tajan et al., Nat Commun. 2021, PMID: 33446657; Yi-Ping Wang et al., Mol Cell. 2016, PMID: 27840030; Tianzhi Huang et al., Mol Cell. 2021, PMID: 33539787). For animal studies, 6 mice per group were used.
Data exclusions	No samples or animals were excluded from the analyses.
Replication	All experiments were repeated independently with similar results for at least 3 times, unless specified. For animal experiment, 5 or 6 mice were used in each group.
Randomization	Mice were randomly allocated to different groups. No randomization was applied on cell-based experiments because there were defined as different groups, e.g., GFP-PRMT1-WT vs. GFP-PRMT1-K37R.
Blinding	The investigators who performed quantification of immunohistochemistry (IHC) analysis were blinded to the patient information. Blinding is not applicable for the in vitro experiments in this paper, because the same investigator performed cell culture, treatments andend-point analysis. Automated quantitative methods were used to eliminate subjective interpretation of data.

Reporting for specific materials, systems and methods

We require information from authors about some types of materials, experimental systems and methods used in many studies. Here, indicate whether each material, system or method listed is relevant to your study. If you are not sure if a list item applies to your research, read the appropriate section before selecting a response.

Materials & experimental systems

n/a	Involved in the study
☐	☒ Antibodies
☐	☒ Eukaryotic cell lines
☒	☐ Palaeontology and archaeology
☐	☒ Animals and other organisms
☒	☐ Clinical data
☒	☐ Dual use research of concern
☒	☐ Plants

Methods

n/a	Involved in the study
☒	☐ ChIP-seq
☒	☐ Flow cytometry
☒	☐ MRI-based neuroimaging

Antibodies

Antibodies used

Anti-PHGDH (mouse, 1:1000), Abcam, cat: ab57030, lot: GR3367647-4;
Anti-GFP (rabbit, 1:5000), Abcam, cat: ab32146, E385, lot: GR253725-18;
Anti-PRMT1 (rabbit, 1:1000 for immunoblotting, and 1:100 for IHC staining), Abcam, cat: ab190892, EPR18344, lot: GR222684-5;
Anti-PRMT1 (rabbit, 1:4000 for immunoblotting), Proteintech, cat: 11279-1-AP, lot: 00118668;
Anti-HA (mouse, 1:4000), Abcam, cat: ab18181, HA.C5, lot: GR3357646-1;
Anti-HA (rabbit, 1:5000), Abcam, cat: ab9110, lot: 1017711-1;
Anti-PHGDH (rabbit, 1:1000), ThermoFisher Scientific, cat: PA5-27578, lot: VI3078259E;
Anti-FBXO7 (rabbit, 1:500 for immunoblotting, and 1:100 for IHC staining), ThermoFisher Scientific, cat: PA5-115219, lot: 33915A03;
Anti-cleaved caspase 3 (rabbit, 1:500 for immunoblotting, and 1:200 for IHC staining), Cell Signaling Technology, cat: 9661, lot: 47;
Anti-caspase 3 (rabbit, 1:1000), ABclonal, cat: A0214, lot: 3560077017;
Anti-8-oxo-dG (Mouse, 1:50 for IHC staining), Abcam, cat: ab206461, lot: 1040855-1;
Anti-GFP (mouse, 1:2000), Roche, cat: 11814460001, lot: 70378300;
Anti-FLAG (mouse, 1:4000), Sigma-Aldrich, cat: F3165, clone M2, lot: SLBT6752;
Anti-FLAG (mouse, 1:100 for immunofluorescence), Sigma-Aldrich, cat: F1804, lot: SLCQ9255;
Anti-Ki-67 (rabbit, 1:200 for IHC staining), Sigma-Aldrich, cat: AB9260, lot: 3857660;
Anti-FBXO7 (mouse, 1:500), Santa Cruz Biotechnology, cat: sc-271763, lot: D0414;
Anti-GST (rabbit, 1:2000), Proteintech, cat: 10000-0-AP, lot: 20000190;
Anti-His (rabbit, 1:4000), Proteintech, cat: 66005-1-Ig, lot: 10027681;
Anti-ubiquitin (rabbit, 1:1000), PTM BIO, cat: PTM-1106RM, lot: RN010622
Anti-β-actin (rabbit, 1:4000), ABclonal, cat: AC026, lot: 9100026001;
The site-specific antibody recognizing mono-methyl R236 of PHGDH (mePHGDH (R236me1), 1:500 for immunoblotting, and 1:50 for IHC staining) was customized from GL Biotech (Shanghai, China).

Validation

All antibodies have been validated on the product webpages or literature, including: Anti-PHGDH (mouse), Abcam, cat: ab57030 (https://www.abcam.cn/phgdhmalate-dehydrogenase-antibody-4a3-1d6-ab57030.html), PMID: 28122957, 23979213, 30348640;
Anti-GFP (rabbit), Abcam, cat: ab32146 (https://www.abcam.cn/gfp-antibody-e385-ab32146.html), PMID: 33976182, 31973889, 32097085;
Anti-PRMT1 (rabbit), Abcam, cat: ab190892 (https://www.abcam.cn/prmt1-antibody-epr18344-ab190892.html), PMID: 33997990, 29794014, 31536826;
Anti-PRMT1 (rabbit), Proteintech, cat: 11279-1-AP (https://www.ptgcn.com/products/ PRMT1-Antibody-11279-1-AP.htm), PMID: 37071992; 36922568; 33420374;
Anti-HA (mouse), Abcam, cat: ab18181 (https://www.abcam.cn/ha-tag-antibody-hac5-ab18181.html), PMID: 33436623, 33950834, 31903784;
Anti-HA (rabbit), Abcam, cat: ab9110 (https://www.abcam.cn/products/
primary-antibodies/ha-tag-antibody-chip-grade-ab9110.html); PMID: 36522330, 35021090, 35177643;
Anti-PHGDH (rabbit), ThermoFisher Scientific, cat: PA5-27578 (https://www.thermofisher.cn/cn/zh/antibody/product/PHGDH-Antibody-Polyclonal/ PA5-27578), PMID: 34720086, 36823188;
Anti-FBXO7 (rabbit), ThermoFisher Scientific, cat: PA5-115219 (https://www.thermofisher.cn/cn/zh/antibody/product/FBXO7-Antibody-Polyclonal/
PA5-115219), validated on the product webpage;
Anti-cleaved caspase 3 (rabbit), Cell Signaling Technology, cat: 9661 (https://www.cellsignal.cn/products/primary-antibodies/cleaved-caspase-3-asp175-antibody/9661), PMID: 37731609, 37739951;
Anti-caspase 3 (rabbit), ABclonal, cat: A0214 (https://abclonal.com.cn/ catalog/A0214), PMID: 34815412, 36882522;
Anti-8-oxo-dG (Mouse), Abcam, cat: ab206461 (https://www.abcam.cn/products/
primary-antibodies/oxoguanine-8-antibody-2q2311-ab206461.html), PMID: 31578304, 28916735;
Anti-GFP (mouse), Roche, cat: 11814460001 (https://www.sigmaaldrich.cn/
CN/zh/product/roche/11814460001); PMID: 34616022, 33030392, 35239512;
Anti-FLAG (mouse), Sigma-Aldrich, cat: F3165 (https://www.sigmaaldrich.cn/CN/zh
/search/f3165?focus=papers&page=1&perpage=30&sort=relevance&term=f3165&type=citation_search), PMID: 31417089, 31365523, 32265302;
Anti-FLAG (mouse), Sigma-Aldrich, cat: F1804 (https://www.sigmaaldrich.cn/CN/zh/
search/f1804?focus=products&page=1&perpage=30&sort=relevance&term=F1804&type=product), PMID: 25697406, 28053121, 35950911;
Anti-Ki-67 (rabbit), Sigma-Aldrich, cat: AB9260 (https://www.sigmaaldrich.cn/CN/zh/

product/mm/ab9260), PMID: 26056141, 30517867, 26795843;
Anti-FBXO7 (mouse), Santa Cruz Biotechnology, cat: sc-271763 (https://www.scbt.com/p/fbxo7-antibody-e-8?requestFrom=search); PMID: 34800438, 36646384, 29103612;
Anti-GST (rabbit), Proteintech, cat: 10000-0-AP (https://www.ptgcn.com/products/gst-Antibody-10000-0-AP.htm), PMID: 36575184, 36436593, 31882361;
Anti-His (rabbit), Proteintech, cat: 66005-1-Ig (https://www.ptgcn.com/products/His-Tag-Antibody-66005-1-Ig.htm), PMID: 32494007, 37480842;
Anti-ubiquitin (rabbit), PTM BIO, cat: PTM-1106RM (http://www.ptm-biolab.com.cn/ productDetail.html?id=9151), PMID: 37858678, 37598917;
Anti-β-actin (rabbit), ABclonal, cat: AC026 (https://abclonal.com.cn/catalog/AC026), PMID: 33603116, 36243008, 32368828; The site-specific antibody recognizing mono-methyl R236 of PHGDH (mePHGDH (R236me1)) was customized from GL Biotech (Shanghai, China). The specificity of this antibody was validated in a previous study (PMID: 36823188).

Eukaryotic cell lines

Policy information about cell lines and Sex and Gender in Research

Cell line source(s)	Huh7, PLC/PRF/5 and HEK293T cells were obtained from the Bank of Type Culture Collection of Chinese Academy of Sciences.
Authentication	All cell linces were authenticated by fingerprinting of short tandem repeats.
Mycoplasma contamination	All cell lines were routinely tested negative for mycoplasma.
Commonly misidentified lines (See ICLAC register)	No commonly misidentified lines were used.

Animals and other research organisms

Policy information about studies involving animals; ARRIVE guidelines recommended for reporting animal research, and Sex and Gender in Research

Laboratory animals	BALB/c nude mice (6-week-old) were used for xenograft studies of HCC cells. Mice were housed under ambient temperature of 24 ± 2 °C, circulating air, constant humidity of 50 ± 10%, and a 12 h: 12 h light/dark cycle.
Wild animals	Not used.
Reporting on sex	No specific consideration was given to mice sex.
Field-collected samples	No field-collected samples.
Ethics oversight	All animal studies were performed in accordance with guidelines provided by the Institutional Animal Care and Treatment Committee of Sichuan University. The animals were treated in accordance with relevant institutional and national guidelines and regulations. The maximal allowable tumor size/burden (diameter≤1.5 cm) was not exceeded. Mice were euthanized by cervical dislocation at the end of the experiment.

Note that full information on the approval of the study protocol must also be provided in the manuscript.
